# Vaccination against HPV: boosting coverage and tackling misinformation

**DOI:** 10.1002/1878-0261.12808

**Published:** 2020-10-15

**Authors:** Janne Bigaard, Silvia Franceschi

**Affiliations:** ^1^ The Danish Cancer Society Prevention & Information Copenhagen Denmark; ^2^ Centro di Riferimento Oncologico di Aviano (CRO) IRCCS Aviano Italy

**Keywords:** cervical cancer, coverage, HPV, misinformation, social media, vaccination

## Abstract

The availability of human papillomavirus (HPV) vaccines and screening tests has raised the possibility of globally eliminating cervical cancer, which is caused by HPV. Cervical cancer is a very common malignancy worldwide, especially among deprived women. High vaccination coverage is key to the containment and eventual elimination of the infection. Public HPV vaccination programmes in Italy and Denmark were swiftly established and are among the most successful worldwide. Still, in both countries, it has been challenging to achieve and maintain the recommended coverage of > 80% in girls. In a well‐studied Italian region, vaccination coverage in girls at age 15 years (World Health Organization's gold standard) reached 76% in 2015 but decreased to 69% in 2018, likely due to work overload in public immunization centres. In Denmark, doubts about safety and efficacy of the HPV vaccine generated a decline in coverage among girls age 12–17, from 80% in 2013 down to 37% in 2015, when remedial actions made it rise again. Insights from these two countries are shared to illustrate the importance of monitoring coverage in a digital vaccine registry and promptly reacting to misinformation about vaccination.

## Graphical abstract

High coverage of human papillomavirus (HPV) vaccination is key to the eventual elimination of infection and cervical cancer. Monitoring coverage, in a digital vaccine registry, is therefore essential to detect problems. For example, misinformation about dangers from HPV vaccine generated in Denmark a decline in coverage among girls age 12–17, from 80% in 2013 down to 37% in 2015, when remedial actions made it rise again.

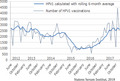

**Abbreviations**
CC: cervical cancer
FVG: Friuli Venezia Giulia
HICs: high‐income countries
HPV: human papillomavirus
LMICs: middle‐income countries
WHO: World Health Organization

## 1 Introduction

Many studies throughout the twentieth century suggested that cervical cancer (CC) was related to a sexually transmitted infectious agent. After considering different ‘candidates’ including herpes simplex virus and *Chlamydia trachomatis*, vast human and molecular evidence led to the recognition of human papillomavirus (HPV) as a necessary although not sufficient cause of CC [1]. HPV was also found to be responsible for a substantial fraction of less frequent tumours of the anogenital tract and oropharynx.

In less than two decades, powerful tools to screen for and prevent HPV infection became available. Three highly efficacious and safe prophylactic vaccines against HPV have been released since 2006, all targeting at least the two most carcinogenic types of the HPV family, that is HPV16 and HPV18 [2]. These two types are responsible for approximately 70% of CC worldwide and nearly all HPV‐related cancers in other sites [3]. Greatest protection against CC is achieved when HPV vaccines are administered prior to sexual debut, as HPV is often contracted by both females and males soon after sexual exposure of any kind [1].

The concurrent availability of vaccines and viral screening tests has raised a real possibility of globally eliminating CC, a very common malignancy among underprivileged women in high‐income countries (HICs) and in the general female population in many low and middle‐income countries (LMICs) [2, 3]. In reality, scaling‐up HPV vaccination programmes has been difficult in most parts of the world [4], including in European countries [5], on account of the relatively high cost of the vaccines and the target age, as adolescent immunization is more challenging than childhood immunization.

Here, we focus on two important issues that can threaten the success of HPV vaccine programmes even in HICs with well‐established and free‐of‐charge immunization programmes run by public national health systems. We will specifically address (a) failure to reach and sustain an adequate coverage, and (b) damage from misinformation about the safety or the efficacy of HPV vaccines. While the Italian case will mainly illustrate the problem of achieving and sustaining high and equal coverage in the target population, the Danish case will be discussed in respect to impact of misinformation about vaccine safety and the subsequent remedial action taken by the Danish Health Authority, the Danish Medical Association and the Danish Cancer Society. We will also emphasize the global importance of these challenges.

## 2 Introduction of HPV vaccination in Italy and Denmark

### 2.1 Italy

The implementation of HPV vaccination in Italy [6, 7, 8] and Denmark [9, 10] has been described elsewhere. Briefly, in Italy, a national HPV vaccination programme started in 2008. It was based on active invitation (by regular mail) of eligible individuals and the free‐of‐charge delivery of vaccine doses in public immunization centres that also distribute paediatric vaccines. Vaccination was initially offered free‐of‐charge to girls aged 11–12 (first birth cohorts: 1997) and girls aged 15(catch‐up). Girls aged 16–18 could get vaccination free‐of‐charge upon request. Boys aged 11–12 were included into the HPV vaccination programme in 2015 (first birth cohort: 2004). The HPV vaccination was later offered free‐of‐charge to women up to 25 years and several categories of high‐risk women and men, for example immunosuppressed individuals.

### 2.2 Denmark

In Denmark, routine HPV vaccination began in 2009 to girls aged 12 (first birth cohort: 1996). At the same time, girls aged 13–15 (born 1993–1995) received a catch‐up offer free‐of‐charge from October 2008 to the end of 2010. Young women born 1985–1992 were offered the vaccine from September 2012 to December 2013 [10]. Danish boys aged 12 (first birth cohort: 2007) were included in the programme in 2019. A catch‐up programme for boys born in 2006 and first half of 2007 was offered from February 2020 to December 2021. Men aged 18 to 25 who have sex with men were offered HPV vaccination free‐of‐charge from February 2020 until the end of 2021 [11]. Initially, parents in Denmark were not actively prompted to have their daughters vaccinated, but they received a reminder from the Statens Serum Institut when a girl turned 14 years. Since the end of 2019, parents have been receiving a warning by electronic e‐mail when their girls or boys turn 12 years. All vaccine doses are provided free‐of charge by general practitioners as part of the National Childhood Vaccination Programme.

In both Italy and Denmark, the number of doses of HPV vaccine recommended in individuals below age 15 were decreased from three to two in 2014, on account of the demonstration of noninferiority [3].

## 3 Coverage of HPV vaccination

### 3.1 Global picture

High coverage is key to the containment and eventual elimination of an infection for which an effective vaccine exists. Coverage does not need to be 100%, but it requires that the vast majority of a population is protected either directly (by being vaccinated) or indirectly (due to the decline of the circulation of an infection following mass immunization). In order to stop the rapid spread of air‐borne infections among entirely susceptible children (e.g. measles), 95% coverage is recommended. For a sexually transmitted virus‐like HPV, adequate coverage may be a little lower at around 80% [12].

**Fig. 1:**
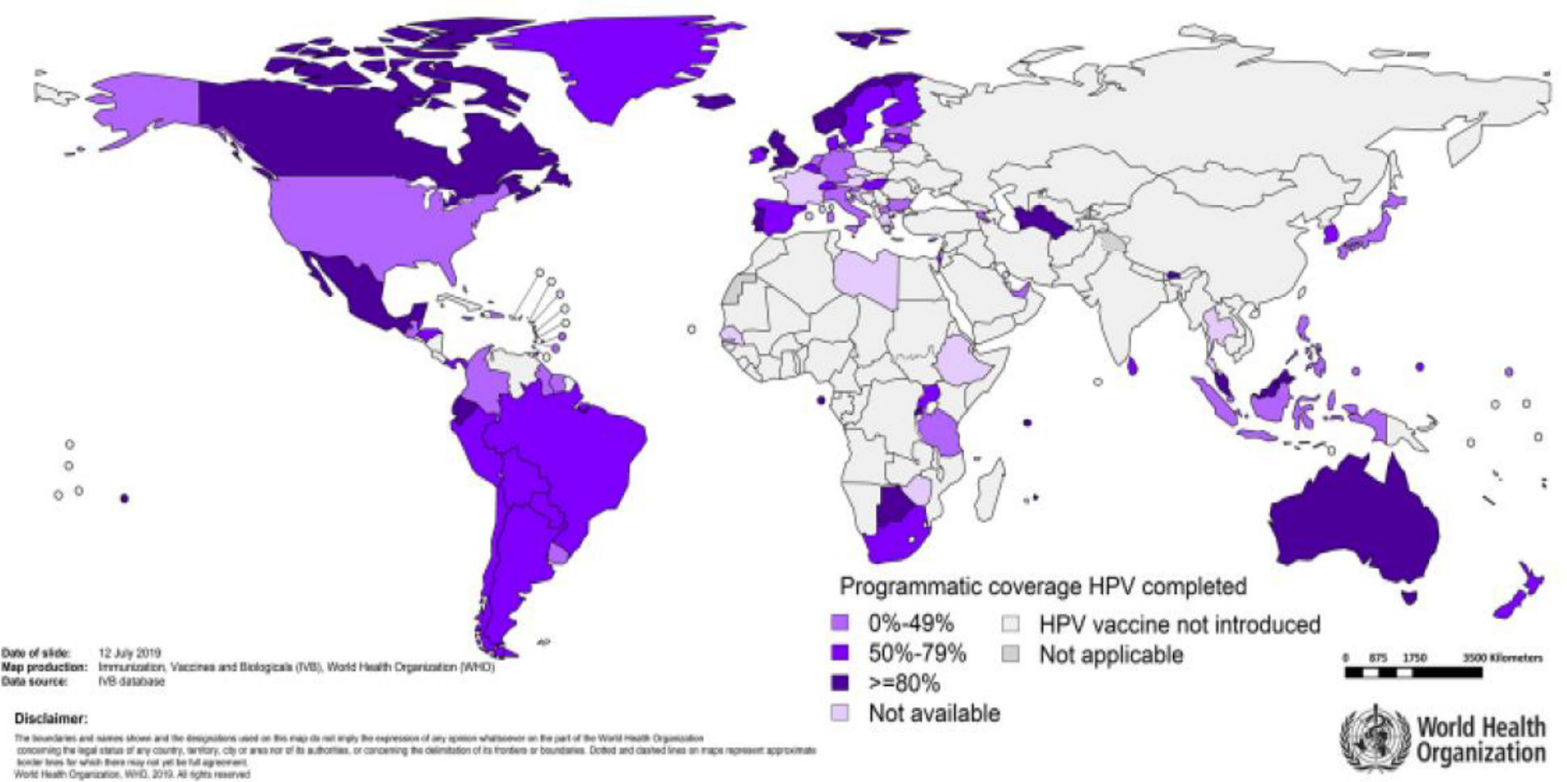
HPV coverage estimates available for 75 member states (Figure reproduced from UNICEF/WHO [13]).

Vaccination for HPV had been introduced in more than 100 countries by the end of 2019. World Health Organization (WHO) surveillance data for 75 countries (Fig. 1) [13] show that coverage was high (≥ 80) in Australia [14], Canada, Latin America and fairly high (about 50%) in the United States [15]. In Africa, Rwanda and South Africa reached ≥ 80 coverage, whereas either no vaccination or no information on coverage is available for the majority of the continent. The picture in Asia is even gloomier, with ≥ 80 coverage in Bhutan, Malaysia and South Korea, but no vaccination programmes available yet in some of the region’s most populous countries including India and China [16]. Interestingly, HPV Vaccine coverage varies substantially regardless of income strata, i.e., HICs or LMICs.

Nearly all European countries quickly launched national HPV vaccination programmes after 2006, but coverage is often unsatisfactory and inconsistently reported, if at all [4, 5]. Full‐course coverage of ≥ 80% among adolescent girls has been attained in the United Kingdom, Sweden and Norway, all of which have opted for school‐based HPV vaccination. Some countries in which HPV vaccine was offered free‐of‐charge but not in schools, like Italy, Portugal and Spain, have achieved around 70% coverage in girls or, in Germany, 50%. Denmark had initially > 80 coverage, but it dropped substantially later on. Conversely, in countries where the HPV vaccine is paid out‐of‐pocket (most Eastern European countries) or using a co‐pay model (e.g. France) coverage has been below 30% [5].

### 3.2 Gold standard for monitoring coverage

Monitoring coverage accurately over time is necessary for sustaining an effective, socially fair and cost‐effective immunization programme. However, monitoring coverage is particularly challenging in the case of HPV vaccination because well‐established delivery and recording systems that include adolescents and adults exist only in very few countries.

The WHO recommends to vaccinate adolescents, primarily girls, between age 9 and 14 (routine immunization) [17]. However, to speed up the effect of the vaccine, catch‐up (also referred to as multi‐cohort) programmes for older girls and young women have been also introduced in many countries since 2006 and so have immunization programmes for boys in HICs since 2013 [5, 8].

National HPV programmes that use call/recall system, for example, those in Italy, Denmark, the UK and Australia, typically invite vaccine‐eligible adolescents by birth cohort and, accordingly, monitoring coverage is most commonly done by birth cohort [7, 8]. When assessing HPV vaccination trends over time, coverage by age and cohort coincide in each specific calendar year, but the correspondence is lost when comparing coverage by cohort in different years. The problem is that HPV vaccine, as a new vaccine, tends to be considered by many families and physicians an ‘optional’ vaccine that can be delayed, the more so in countries like Italy and Denmark, where HPV vaccine is offered for free also to older girls and young women. Coverage in a specific birth cohort can therefore increase year after year, and lower coverage in the youngest birth cohorts can be due to either a decline or the tendency to delay vaccination.

For this reason, WHO recommends monitoring coverage in adolescents up to the day preceding their 15^th^ birthday (hereafter referred to as ‘at 15’), assuming that each targeted birth cohort has had the opportunity to complete vaccination by that age [17]. Only Australia [14] and a few countries in Europe [5] that have opted for school‐based vaccination have been able to comply with this monitoring system because this indicator requires individual information on all the doses of vaccine administered prior to age 15, which is a difficult task without a digital vaccination registry.

### 3.3 Coverage at age 15, the example of Italy

**Fig. 2:**
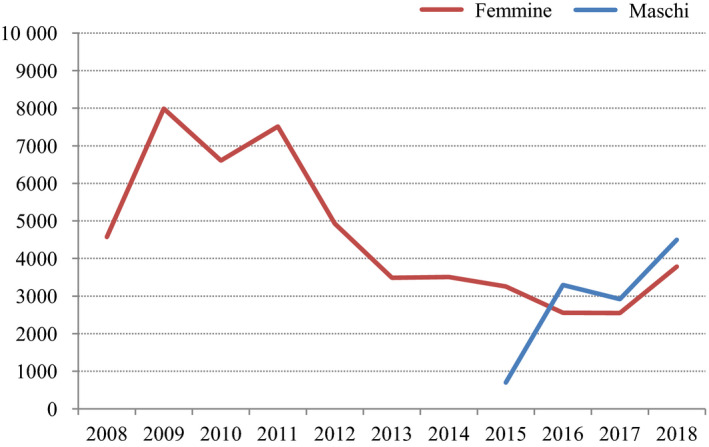
Number of girls and boys aged 11–18 receiving ≥ 1 dose each year, FVG Region, North‐Eastern Italy, 2008–2018 (Figure modified from [8]).

The following data offer an example of the usefulness of monitoring coverage at age 15 based on data from Friuli Venezia Giulia (FVG), a region in North‐eastern Italy with about 1.2 million inhabitants, excellent health‐quality indicators, and a good digital vaccination registry[8]. By the end of 2018, 52 217 females (from 2008) and 12 152 males (from 2015) had been vaccinated. The number of girls and boys aged 11–18 who received ≥ 1 dose each year is shown in Fig. 2. Approximately 8000 girls per year were initially vaccinated but the number fell to less than 4000 in 2013 when the catch‐up programme nearly stopped. The addition of boys to the programme in 2015 gradually increased again the number of vaccinated adolescents, that is 8000 in 2018, half for each gender (Fig. 2).

Full‐course (≥ 2 doses) coverage in girls at age 15, however, reveals a different picture. Coverage rose from 42% in 2009 to 72% in 2012 and 76% in 2015 but declined to 69% in 2018 [8]. Birth cohort analyses in girls (updated at 31 December 2017) in the FVG region would suggest instead a constant decline in coverage from 71% in girls born in 1997 to 67% in those born in 2003 down to 42% in the 2005 cohort [8]. Birth cohort‐based coverage in this region was similar to the trend seen in the rest of Italy [6].

### 3.4 Safety data on HPV vaccination

All HPV vaccines consist of highly purified virus‐like particles that consist of protein shells of the HPV virus. They contain no viral DNA and, therefore, cannot cause infections or disease. In addition, the safety of HPV vaccine has been thoroughly evaluated in clinical trials and in population surveillance programmes [18] and has been the object of extensive systematic reviews demonstrating reassuring safety profile for bivalent, quadrivalent [19] and nonavalent vaccines [20]. Postlicensure surveillance data have detected the occurrence of site‐related pain, swelling and erythema but no serious safety issues to date, except rare reports of anaphylaxis (1.7 cases per million doses). Postural orthostatic tachycardia syndrome and chronic regional pain syndrome have been reported as adverse events following HPV immunization, but these conditions can rarely occur regardless of immunization and their pathogenesis is poorly understood. Their association has been reviewed by the European Medicines Agency who have concluded that there is no causal association between these conditions and HPV immunization [21].

## 4 Tackling misinformation

### 4.1 The Danish story

#### 4.1.1 Mistrust

More than 80% of Danish girls were fully vaccinated when the HPV vaccine was first introduced in 2009 [9]. In 2013, however, the media started reporting personal stories from girls and young women who experienced unspecified symptoms sometime after their HPV vaccination [9, 10]. In 2015, national television broadcasted a documentary called ‘The vaccinated girls’ that suggested a causal relationship between the reported unspecific symptoms and the HPV vaccine [22]. Immediately after, Statens Serum Institut (SSI) reported a steep decline in HPV vaccination uptake although no evidence had showed the vaccine to be unsafe [9]. This situation in Denmark was unique among the Nordic countries, but other countries including Ireland, France, Romania [23], Colombia [24] and Japan [25] also experienced similar cases of mistrust specifically against the HPV vaccine. Danish health professionals tried to restate vaccine safety using fact‐based communication but did not succeed in restoring vaccination uptake. The Danish Health Authority therefore invited the Danish Medical Association and the Danish Cancer Society to cooperate and start a brand‐new collaborative information campaign to regain trust in HPV vaccination.

The WHO describes vaccine mistrust as a growing gap emerging between experts and citizens [26]. In Denmark, medical doctors, researchers and health authorities who tried to communicate the safety of the vaccine using facts tend to be perceived by many citizens as arrogant. In 2016, Danish parents were still afraid that the potential harms induced by HPV vaccination might be worse than the disease it could prevent [27]. What they needed to diminish their worries was emotionally based communication that combined personal stories about CC with facts about HPV vaccine [28].

#### 4.1.2 Remedial campaign

A joint information campaign launched in 2017 aimed at preventing CC by increasing the uptake of HPV vaccination within the Danish Childhood Vaccination Programme. Extensive preparations preceded a collaborative communications strategy, which combined targeting the press, the social media, doctors and other health professionals, and parents in order to foster an informed decision about HPV vaccination [29].

An important realization arising from these pre‐analyses was that mothers most often decided about vaccination in Denmark [28]. Many mothers were therefore involved in focus‐group interviews to provide insights into their knowledge and the barriers to HPV vaccination, notably the lack of clear information and recommendations about HPV vaccination from health authorities and other organizations they trusted. For further support, a broad network of stakeholders, including professional doctor and nurse organizations or societies, sexual health organizations and patient cancer associations, was involved to back up the launching of the campaign [29].

The communications strategy evolved as described by the WHO: through the definition of the target group and its segments; the understanding of the barriers and motivations of the target group; and the preparation of distinct messages and activities for the various segments of the target group [26]. An earlier Facebook site communicating about CC screening and HPV vaccination became valuable during the preparation of a Question and Answer (Q&A) guide. The questions and comments in the threads of Facebook site helped develop answers directed to both the’brain’ and the ‘heart’, including facts and personal stories.

However, this Facebook account was inactivated at a later stage, as it had become dominated by users spreading misinformation [29]. Nevertheless, the information campaign had to be visible at platforms were mothers seek information about HPV vaccination, and Facebook was specified as their primary channel of information [28]. Social media platforms offered a space for dialogue, where mothers in doubt could ask questions and receive answers to facilitate informed decision‐making. Personal stories from CC survivors, women with cervical intraepithelial neoplasia or parents in doubt who reached a decision to vaccinate became valuable ‘heart‐stories’ [28]. Conversely, Instagram was essential to reach girls between 15 and 17 years, who were still eligible for free HPV vaccination [29]. Notably, substantial manpower was essential to answer parents’ questions and to prevent misinformation from dominating the digital channel through daily monitoring and prompt responses. Students were, therefore, usefully employed to monitor Facebook pages with around‐the‐clock access to experienced campaign staff [29].

#### 4.1.3 Outcomes

**Fig. 3:**
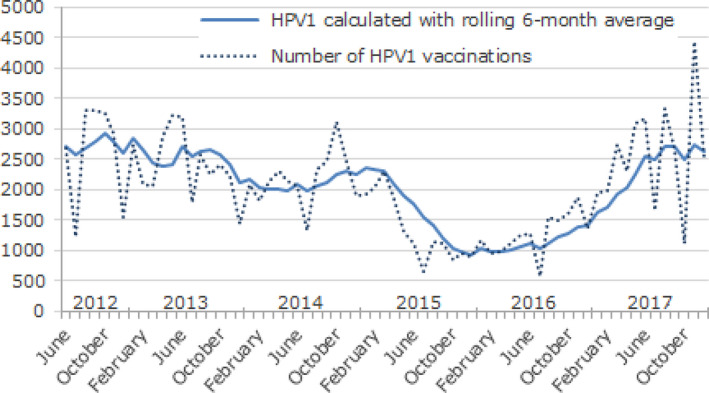
Number of young women aged 12–17 who initiate HPV vaccination per month, June 2012 to December 2017 (Figure reproduced from Statens Serum Institut [30]).

Within the first year of the campaign, and as a result of a more balanced communication through the press, voices criticizing HPV vaccination became less dominant [10, 22]. Figure 3 shows that the steep decline in HPV vaccination after the broadcast of the television documentary was followed by a doubling in HPV vaccine uptake in 2017; a further 20% increase in 2018; and a rise to 36 560 girls vaccinated in 2019 that exceeded the approximate count of a birth cohort, that is 33 000 girls [30]. The percentage of Danish parents who were in doubt about vaccination (38% before the remedial campaign) diminished to 14% in 2019 and even the parents who completely rejected vaccination decreased from 16% to 5% (Bigaard J, personal communication). The proportion of parents who had heard negative stories about HPV vaccination in the media also decreased. However, half of parents in doubt continued to be worried about possible serious adverse side of HPV vaccination.

At the same time, the media debate had become more impartial and supportive of the campaign against misinformation. Indicatively, an independent editor who oversees the interest of the TV viewers rated the aforementioned TV documentary as partial by focusing on girls with unspecified symptoms and not the evidence [31]. Likewise, a Danish Fact‐checking website was launched in 2019 in the framework of the International Fact‐checking Network (IFCN) [32]. The number of HPV vaccinations increased steadily and 72% of Danish girls (born 2002–2006) were fully vaccinated in 2020 [33]. However, the country is not yet back to the previous high level of 80%. Notably, the hesitancy and misinformation that hit HPV vaccination did not affect paediatric vaccines whose uptake is above 90%.

### 4.2 The Italian story

Italy was less affected by mistrust in the HPV vaccine than Denmark. There were initially fears about the issue of vaccinating young girls s against a sexually transmitted disease, but the topic was never really in the spotlight. Some catholic organizations even supported the programme, and there is some evidence that migrants of different ethnic groups participated as often as Italian adolescents [8]. In general, paediatricians and gynaecologists were either supportive or neutral. By comparison, the nearly contemporary gradual substitution of Pap smear with HPV testing in the framework of the organized cervical screening had been accompanied by much harsher public debate [34]. Initial hostility by some gynaecologists and cytologists against the shift to HPV testing was eventually superseded in Italy by the demonstration of superior cost‐effectiveness [35] and the growing recognition that cancer screening as well as vaccination must be guided by public health principles and not by private medicine’s views. The Ministry of Health, regional authorities and scientific societies launched initiatives in support of HPV vaccination such as Q&A guides [36]. However, vaccine manufacturers represented the most vocal source of advocacy in Italy and may have contributed to the rapid broadening of the free‐of‐charge offer of HPV vaccination to population subsets other than adolescents, including various categories of high‐risk women and men of any age despite the lack of clear scientific evidence of efficacy of HPV vaccination among high‐risk adults.

Conversely, and contrary to what had happened in Denmark, in the 2010s misinformation and probably intentional fake news in Italy hit hard paediatric vaccinations. False beliefs and conspiracy theories spread on the Internet, and an antivaccination sentiment infected not only parents of small children but also celebrities and politicians [37]. After rising for years, measles vaccination uptake in Italy started declining and they recovered only after a capillary public re‐education campaign and the approval of a law that made 10 paediatric vaccines mandatory again [38].

## 5 Discussion

Human papillomavirus vaccine coverage among girls at age 15 in Italy and Denmark is presently among the highest in Europe and worldwide [5]. Nevertheless, even in FVG, one of the best‐organized Italian regions, the lack of improvement in girls’ HPV vaccination coverage in the last years [8] is of concern. It may be a sign of ‘programme fatigue’ consequential to the increase in tasks given to public immunization centres, including catching‐up with children who had been missed in the years in which misinformation against paediatric vaccines had spread [38]. By and large, public health investment in Italy stagnated between 2007 and 2016 and school‐based health services had long disappeared [39]. The learning is therefore that new vaccination tasks cannot be lightly assigned to immunizations centres without factoring in the need to improve their resources and manpower. This caveat is even more essential when introducing new vaccination schemes in LMICs. In respect to HPV vaccination, the inclusion of adolescents of both sexes in Italy and elsewhere can speed up the beneficial impact of the intervention [40, 41], but it should not subtract energy from the most cost‐effective target group, namely girls before sexual debut [42].

Conversely, HPV in Denmark vaccination was mainly threatened by a wave of misinformation that hit an otherwise well‐working HPV vaccination programme. Professionals may not fully appreciate the severe impact of modern sources of misinformation, including the social media, were fake news spread faster than the truth [43]. Unfortunately, scientists do not resonate as well with the public as an alarm or other statements made by a celebrity [44]. In the Danish case that we have described, it was the people who spread misinformation and, possibly, even malicious fake news rather than robots, that is computer‐led machines capable of multiplying messages in social media. However, increasing use of robots may worsen the problem [43]. Health professionals should therefore acknowledge the threat and engage with social media [23, 44], especially following the revelation that mothers who declined HPV vaccination sought information on social media rather than from their general practitioner [45]. Indeed, Betsch *et al*. [46] showed that accessing vaccine‐critical websites increases the perception of the risk of vaccinating and decreases the perception of the risk of omitting vaccinations.

The learning from Denmark has been to never leave unanswered questions and to keep in mind the silent audience. Without brave women sharing their personal stories about their lives with CC or intraepithelial dysplasia, the information campaign would be less effective [28].

## 6 Conclusion and perspectives

This review emphasizes the need for standardized methods to monitor HPV vaccination nationally and internationally. While coverage by birth cohort is commonly used, we echo WHO’s plea to also measure coverage in girls at age 15 as an indicator of not only uptake but also of timeliness of HPV vaccination. Prioritizing HPV vaccination of girls may become imperative in the COVID‐19 era, during which preventative activities are under added pressure in many countries (https://www.who.int/news‐room/detail/15‐07‐2020‐who‐and‐unicef‐warn‐of‐a‐decline‐in‐vaccinations‐during‐covid‐19). If vaccine mistrust arises, the existence of valuable monitoring tools will help document drops in a programme performance as well as re‐establishing confidence following a crisis. Misinformation can make considerable damages and, therefore, scientists, health workers and media should gather to provide appropriate communication directed to both the ‘brain’ using facts and the ‘heart’ using personal stories.

## Conflict of interest

The authors declare no conflict of interest.

## Author contributions

JB and SF equally contributed to the manuscript. JB reviewed the Danish data and SF the Italian data. JB and SF both approved the final paper.
